# Impact of Autonomy Support on the Association between Personal Control, Healthy Behaviors, and Psychological Well-Being among Patients with Hypertension and Cardiovascular Comorbidities

**DOI:** 10.3390/ijerph19074132

**Published:** 2022-03-31

**Authors:** Hyun-E Yeom, Jungmin Lee

**Affiliations:** 1 Department of Nursing, Chungnam National University, Munhwaro 266, Junggu, Daejeon 35015, Korea; yeom@cnu.ac.kr; 2 Korea Research Institute for Vocational Education & Training, Social Policy Building, Sejong National Research Complex, 370 Sicheong-daero, Sejong-si 30147, Korea

**Keywords:** internal–external control, personal autonomy, health personnel, health behavior, quality of life, hypertension, comorbidity

## Abstract

A sense of control and autonomy are key components in guiding health-related behaviors and quality of life in people with chronic diseases. This study investigated whether autonomy support from health professionals moderates the impact of personal control on psychological well-being through healthy behaviors in patients with hypertension and cardiovascular comorbidities. Data from 149 hypertensive patients with comorbid cardiovascular risk factors were collected via self-administered surveys. A moderated mediation effect of a hypothesized model was analyzed using the PROCESS macro bootstrapping method. Autonomy support from health professionals moderated the relationship between personal control and healthy behaviors (B = 0.16, *t* = 2.48, *p* < 0.05), showing that the effect of personal control on healthy behaviors differed by the level of autonomy support. Additionally, autonomy support moderated the mediation effect of healthy behaviors in the relationship between personal control and psychological well-being (Index = 0.15; 95% CI = 0.010, 0.335). The mediation effect existed only in patients with higher autonomy support. The findings demonstrate that autonomy support from health professionals plays a crucial role in reinforcing the positive impact of personal control on healthy behaviors and psychological well-being. Enhancing the supportive attitudes of health professionals that facilitate patients’ autonomous self-regulation is necessary for better health outcomes in people with combined cardiovascular diseases.

## 1. Introduction

Hypertension is a pre-existing representative condition of cardiovascular disease that often exists with other cardiovascular risk factors, such as diabetes and obesity [1]. Notably, recent studies have demonstrated that the coexistence of cardiovascular diseases increases vulnerability to infectious diseases, such as severe acute respiratory syndrome (SARS) and coronavirus disease (e.g., COVID-19) [2,3], highlighting the importance of self-care and therapeutic adherence among individuals with hypertension. However, global data have shown that uncontrolled hypertension related to inactive behavioral management remains a worldwide concern [1], and in Korea, morbidity due to hypertension has barely decreased over several decades [4]. Furthermore, empirical studies have found that patients with hypertension reported more psychological distress than did those with other cardiovascular problems [5]. A large body of health literature has demonstrated the benefits of healthy behaviors in preventing physiological aggravation of disease and psychological distress among populations with various cardiovascular risk factors. Therefore, understanding the factors related to healthy behaviors is crucial to prevent disease-related distress and to maintain psycho-emotional health among hypertensive patients with comorbid conditions.

A substantial number of cognitive-behavioral studies have demonstrated the crucial role of cognitive components in initiating and maintaining health-related behavior and outcomes. Specifically, “personal control,” which represents the internalized view of the possibility of treating and controlling a disease [6,7], has been noted as a cognitive factor that affects how individuals with health threats interpret a problem, expect related consequences, and facilitate executive ability, eventually leading to behavioral and health outcomes [7,8,9]. For example, personal control is associated with various health-promoting and preventive behaviors, such as intake of prescribed medication, diet regulation, and smoking and alcohol abstinence in people with diverse chronic diseases [10]. In addition, many studies have shown that personal control is related to psychological health by affecting the regulation of emotions and thoughts arising from the process of dealing with a stressful situation [7,9,10].

Self-determination theory focuses on basic psychological needs in terms of autonomy, competence, and relatedness as core components that affect motivation and behavioral regulation to achieve a goal [11]. In particular, the healthcare literature has highlighted the crucial role of autonomy in facilitating motivation and behavioral self-regulation among individuals with health problems [12,13]. Empirical studies have shown that autonomy is modifiable, primarily based on interpersonal experience [14,15], and particularly that interaction with health professionals in medical contexts is key to forming autonomy to cope with an illness [15,16]. For example, patients who experienced greater autonomy-supportive attitudes from health professionals exhibited greater compliance with treatment regimens and experienced less psychological burden (i.e., depression and anxiety) [15,16].

**Figure 1:**
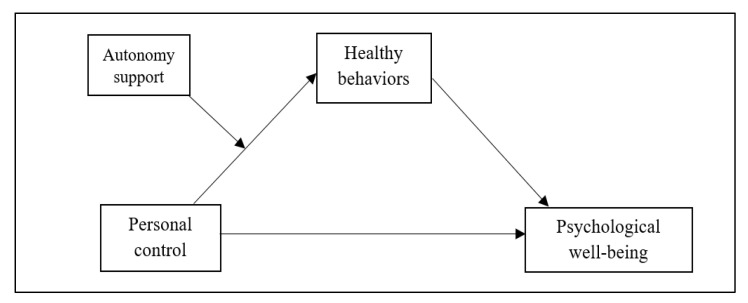
Hypothesized association between personal control, healthy behaviors, and psychological well-being, linked to autonomy support.

A large body of empirical studies have indicated that interactive experiences with health professionals are an essential opportunity for patients to construct their views of diseases, including a sense of control and confidence [12,15]. There is strong evidence for the critical role of personal control and autonomy in guiding health-related behaviors among patients with chronic diseases [7,8,12,14]. However, little is known about the complex relationship between personal control, healthy behaviors, and psychological well-being, given the possible impact of autonomy support from health professionals. Given the principles of self-determination theory, the experience of autonomy-supportive attitudes of health professionals could play an essential role in how personal control affects healthy behaviors and health-related outcomes. Therefore, this study investigated whether the process by which personal control affects psychological well-being through healthy behavior differs depending on the level of autonomy support received by patients with hypertension and comorbid cardiovascular risk factors (see Figure 1).

## 2. Materials and Methods

### 2.1. Samples and Procedure

This was a cross-sectional study. Participants for this study were recruited through convenience sampling in the medical outpatient areas of general hospitals and local clinics in Daegu, Daejeon, and Gyeongbuk provinces of South Korea.

The inclusion criteria for participation in this study were as follows: individuals who (1) were clinically diagnosed with hypertension, (2) had another risk factor for cardiovascular disease (i.e., abdominal obesity, type 2 diabetes, hyperlipidemia, dysrhythmia), and (3) had not been clinically diagnosed as having cognitive dysfunction (i.e., dementia and mild cognitive impairment). In contrast, the exclusion criteria were as follows: individuals who had (1) experienced a severe critical episode of cerebro-cardiovascular diseases (e.g., myocardial infarction and stroke) and (2) other chronic and severe health problems (e.g., chronic obstructive pulmonary disease, chronic kidney dysfunction, cancer).

Data were collected through self-administered surveys completed in the presence of a research assistant from 1 February to 16 April 2021. Three individuals, including one with thyroid cancer history and two with chronic kidney dysfunction, were excluded during the recruitment screening based on the exclusion criteria. Eligible individuals who voluntarily agreed to participate in the study then individually completed a structured questionnaire. If a participant had any queries or difficulties understanding the questionnaire items, research assistants who were graduate students in the nursing program and trained by a primary investigator regarding the recruitment procedure offered their assistance.

The required sample size for the current study was calculated using the program G*Power 3.1.9.7 [17]. Given the conditions of a type 1 error (alpha) of 0.05, medium effect size (0.15) [18], power (1-β error probability) of 0.90, and eight predictors, a minimum of 134 samples was required. Therefore, expecting an approximately 10% drop-out rate and possible missing values in the completed questionnaires, the target recruitment sample size was 150.

Ethical approval for this study was obtained from the institutional review board of the primary researcher’s institution of affiliation (No. 202101-SB-005-01). The purposes and procedures of this study conformed to the principles outlined in the Declaration of Helsinki. The rights guaranteed to participants and procedures for human protection were explained face-to-face and with documents approved by the institutional review board. After receiving and understanding the provided information, potential participants endorsed written consent forms that indicated their voluntary participation in the study.

### 2.2. Measures

The cognitive component of the personal control of disease was assessed using the control subscale of the revised Illness Perception Questionnaire (IPQ-R) [19]. The IPQ-R comprises seven subscales that assess cognitive-emotional perspectives about a disease (i.e., timeline, personal control, treatment control, consequences, predictability, coherence, and emotion), which is concordant with Leventhal’s self-regulatory model [9]. The domain of personal control consists of six items answered on a 5-point Likert scale (1 = strongly agree to 5 = strongly disagree) that assess the extent to which individuals recognize the capability and confidence to control and regulate a health threat. Several items are reverse coded. The average score of the items is calculated, with a higher score indicating greater confidence in controlling and dealing with a disease. The internal consistency of the items in this study was Cronbach’s alpha 0.814.

Autonomy support received from health professionals was measured using the Health Care Climate Questionnaire (HCCQ) [20]. The HCCQ assesses how strongly individuals perceive autonomy support during interactions with health professionals. It comprises 15 items that evaluate attitudinal and behavioral characteristics during medical interactions, such as having an opportunity to express an opinion and ask questions, being asked about choices, and being trusted and respected. Each item was rated on a 7-point Likert scale (1 = strongly disagree to 7 = strongly agree). The level of perceived autonomy support was evaluated by averaging the score of the items, with higher scores indicating greater perceived autonomy support. The internal consistency of the items in this study was Cronbach’s alpha 0.951.

The execution of healthy behaviors to cope with health threats was measured using the Korean version of the Health Behavior Scale [21]. This scale is considered a valid measure to assess the aspects of behavioral practice that individuals with cardiovascular risk perform to prevent complications and promote health in daily living [22]. It comprises 25 items related to physical exercise, diet regulation, abstinence from smoking and drinking alcohol, stress management, and effort to seek health-related information. Each item is rated on a 4-point Likert scale (1 = never to 4 = routinely). The average score of all items was calculated, with higher scores indicating greater proactiveness in engaging in healthy behaviors. The internal consistency of the items in this study was Cronbach’s alpha 0.853.

Psychological well-being was assessed using the Affect Balance Scale (ABS) [23]. The ABS considers positive and negative affective states that reflect life satisfaction and overall happiness. The scale comprises 10 items regarding whether or not an individual has felt certain affective states in the past few weeks, with 5 items measuring positive affect and 5 items measuring negative affect. Each item is scored as 0 if the participant reported not experiencing the given affective state, or 1 if they did experience the affective state. The overall score of affective balance between negative and positive affect was calculated by subtracting the total negative affect score from the total positive affect score. Higher scores indicated greater happiness and life satisfaction. The Kuder–Richardson Formula 20 score was 0.741.

General socio-demographics (i.e., age, gender, job, living status, educational attainment, and household income level) were assessed as covariates potentially related to the main variables. The health-related characteristics, type of cardiovascular comorbidity, and duration since hypertension diagnosis were evaluated. General health status was assessed with a single item, using a 5-point Likert scale (1 = poor, 2 = fair, 3 = good, 4 = very good, 5 = excellent).

### 2.3. Statistical Analyses

Data were analyzed using SPSS (version 26.0; IBM Corp., Armonk, NY, USA). Preliminary analyses to screen the data distribution (i.e., skewness, kurtosis, and outliers) were conducted for the main study variables (i.e., personal control, autonomy support, healthy behavior, and psychological well-being). Descriptive statistics (mean (M), standard deviation (SD), minimum and maximum values, frequency, and proportion) for all study variables were calculated to screen the overall characteristics of the data. The internal consistency of the scales for the main study variables was computed using Cronbach’s alpha coefficients and the Kuder–Richardson Formula 20. Pearson’s correlation coefficients were calculated to determine covariates. Our hypothesized model, which included moderation and mediation effects (see Figure 1), was tested using a bootstrap method of the PROCESS macro for SPSS [24]. A bootstrapping method allows for the resampling of a large number of small samples and reduces the risk of type 1 error, increasing the statistical power [24]. The PROCESS macro analysis tests the moderation and mediation effects of one or more variables on the relationships between the independent and dependent variables within the same model. Specifically, our hypothesized model was analyzed using the PROCESS macro model 7, which calculates a moderation effect, mediation effect, and a moderated mediation effect simultaneously for the whole model. The PROCESS macro was executed with an independent variable (personal control), one mediator (healthy behaviors), a dependent variable (psychological well-being), and one moderator (autonomy support from health professionals). In this study, we applied 5000 bootstrap samples and determined the mediation and moderated mediation effects to the 95% confidence interval (CI).

## 3. Results

### 3.1. Descriptive Characteristics of the Participants

A total of 154 participants completed the self-administered survey and the response rate was 96.8%. The survey data of five respondents were excluded from analyses because the participants did not respond to all the main items of this study. Thus, the data from 149 respondents were analyzed in this study.

The average age of the participants was 60.2 years (SD = 9.0, range = 38–79). More than half of the participants (56.4%) were female, and the female participants (M age = 62.3, SD = 8.6) were on average older than the male participants (M age = 57.9, SD = 8.9). Over half of the participants (*n* = 95, 63.8%) attained an education level of high school graduation and 54 participants (36.2%) were college graduates. The majority of the participants (86.6%) lived with family members, mainly a spouse and children (*n* = 76, 51.0%) or with a spouse only (*n* = 31, 20.8%). Approximately 65% of the participants had a job, and 40.3% of the participants reported their monthly household income as ≥3,000,000 Korean Won (KW), with the median household income being 2–3,000,000 KW.

Regarding health-related characteristics, participants rated their general health status as 2.8 out of 5, which is lower than the median of 3. The average number of years living with hypertension was 6.9 (SD = 6.6). The most common cardiovascular comorbidity among the participants was hyperlipidemia (43.6%), and 23% of the patients had multiple comorbidities (i.e., diabetes and hyperlipidemia). The demographic characteristics are also presented in Table 1.

### 3.2. Reciprocal Relationships between the Main Study Variables

Descriptive statistics for the main study variables and their correlation coefficients are shown in Table 2. The mean scores of the main variables including autonomy support from health professionals, personal control, healthy behaviors, and psychological well-being were 4.90 (SD = 1.10, range = 1.70–7.00), 3.49 (SD = 0.56, range = 1.86–5.00), 2.62 (SD = 0.47, range = 1.40–3.95), and 1.63 (SD = 2.50, range = −5.00–5.00), respectively.

Autonomy support was significantly positively related to healthy behaviors (r = 0.294, *p* < 0.001). Personal control was weakly correlated with healthy behaviors (r = 0.145, *p* = 0.077) but strongly correlated with psychological well-being (r = 0.416, *p* < 0.001). Healthy behaviors, which was considered as a potential mediator in the present study, was positively correlated with psychological well-being (r = 0.239, *p* < 0.01).

### 3.3. Impact of Autonomy Support on the Relationship between Personal Control, Healthy Behaviors, and Psychological Well-being

The results of our hypothesized model (see Figure 1) are shown in Table 3. The model included the moderation effect of autonomy support on the relationship between personal control and healthy behaviors, and the mediation effect of healthy behaviors on the relationship between personal control and psychological well-being according to the level of autonomy support. The PROCESS macro analysis tested the moderation, mediation, and moderated mediation effects simultaneously. The results are presented in the following order: the moderation effect of autonomy support on the link between personal control and healthy behaviors, the mediation effect of healthy behaviors on the relationship between personal control and psychological well-being, and the difference in the mediation effect according to the level of autonomy support (moderated mediation effect).

Table 3 presents the interaction of personal control and autonomy support in predicting healthy behaviors, indicating the moderation effect of autonomy support in the relationship between personal control and healthy behaviors. The covariates for testing our hypothesized model were determined based on having a significant correlation coefficient with psychological well-being, and included general health status (r = 0.290, *p* < 0.001), duration living with hypertension (r = −0.272, *p* < 0.001), age (r = −0.222, *p* = 0.007), and job status (r = 0.214, *p* = 0.009). After adjusting for these covariates, a significant interaction between personal control and autonomy support was found in predicting healthy behaviors (B = 0.16, SE = 0.06, *t* = 2.48, *p* < 0.05). The finding indicates that autonomy support from health professionals moderates the relationship between personal control and healthy behaviors.

**Figure 2:**
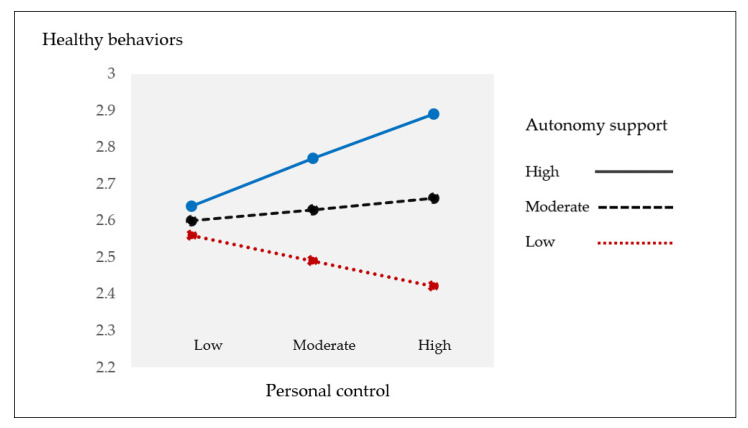
Comparisons of the relationships between personal control and healthy behaviors according to the level of autonomy support.

Figure 2 displays the different patterns in the relationship between personal control and healthy behaviors according to the level of autonomy support. As shown, the impact of personal control on healthy behaviors was remarkably positive within the participants who reported a higher level of autonomy support than those who reported a moderate or lower level of autonomy support.

Table 3 also displays the indirect effect of personal control on psychological well-being through healthy behaviors according to the level of autonomy support (i.e., low, moderate, high). After adjusting for covariates, both personal control (B = 1.48, *p* < 0.01) and healthy behaviors (B = 0.96, *p* < 0.05) were significant predictors of psychological well-being. However, the indirect effect of personal control on psychological well-being through healthy behaviors significantly differed by the level of autonomy support. The indirect effect was significant only among participants who had experienced a higher level of autonomy support (effect = 0.22; SE = 0.12; 95% CI = 0.018, 0.475), not in those who reported a moderate or lower level of autonomy support. Finally, the conditional indirect process that integrates moderation and mediation effects was significant (Index = 0.15; SE = 0.08; 95% CI = 0.006, 0.335), indicating that autonomy support moderates the mediation effect of healthy behaviors in the relationship between personal control and psychological well-being.

## 4. Discussion

Personal control and autonomy have been noted as critical cognitive factors that drive health-related behavior and health outcomes among individuals with chronic health issues. However, little is known about whether health professionals’ autonomy-supportive attitudes affect patients’ cognitive-behavioral processes, specifically regarding the impact of personal control on health-related behaviors and outcomes. Therefore, the current study investigated the role of autonomy support in moderating the process of recognizing that personal control affects psychological well-being through healthy behaviors.

As a key finding, this study delineated an integrated cognitive-behavioral relationship, focusing on the interaction between personal control and autonomy support in predicting healthy behaviors and health outcomes. We found that the perception of personal control affected psychological well-being through healthy behaviors, indicating that healthy behaviors mediated the relationship between personal control and psychological well-being. Notably, we found that the linkage between personal control, healthy behaviors, and psychological well-being differed according to the level of autonomy support received from health professionals. The findings indicate that autonomy support from health professionals played a role as a moderator in the process whereby patients’ perception of personal control affected psychological well-being through healthy behaviors. Regarding the role of autonomy support in regulating the impact of personal control on healthy behaviors and psychological well-being, we discuss the following essential points.

Primarily, we found that the perception of personal control was an essential predictor of healthy behaviors and psychological well-being. The findings align with empirical evidence highlighting a sense of control as a decisive cognitive factor in behavioral practice [8,10] and health outcomes [8,9,10]. It is remarkable that the relationship between personal control and healthy behaviors differed by the level of autonomy-supportive attitudes received from health professionals. In other words, the positive impact of personal control on healthy behaviors tended to be robust when patients received greater support for autonomous self-regulation from health professionals. According to empirical findings, health providers’ attitudes are involved in the cognitive-behavioral processes of patients who are dealing with their health problems [12,13,14,15,16,25]. Concordant with the evidence, this study demonstrated that health professionals’ attitudes in supporting patients’ autonomy contributed to reinforcing the positive impact of personal control on healthy behaviors. Therefore, the findings highlight that health professionals need to recognize the importance of autonomy-supportive attitudes and thereby, interact with patients in a manner that facilitates the autonomy of patients. In addition, the current study found the positive impact of personal control on psychological well-being. The finding aligns with previous findings reporting that stronger beliefs about personal control contribute to enhancing positive affective status [8,10].

Further, we found that personal control indirectly affected psychological well-being through healthy behaviors, which indicates healthy behaviors mediated the relationship between personal control and psychological well-being. The finding implies that psychological well-being is intimately affected by healthy behaviors, rather than being solely dependent on personal control. Beyond extant evidence [26,27], the finding in this study emphasized the importance of promoting healthy behaviors for better health outcomes, by demonstrating its role in mediating the relationship between personal control and psychological well-being.

More interestingly, the present study demonstrated that autonomy support from health professionals moderated the role of healthy behaviors mediating the link between personal control and psychological well-being. That is, the mediating role of healthy behaviors differed by the level of autonomy support and the impact was shown only in patients who had reported a higher level of autonomy support from health professionals, not in those who had reported lower and moderate levels of autonomy support. The finding implies a pivotal role of autonomy support from health professionals in regulating the impact of patients’ personal control on psychological well-being through healthy behaviors. A large body of health literature has indicated that expressing concerns, desires, and opinions during interactions with health professionals is an essential opportunity for patients to recognize and activate autonomy in the process of dealing with their health problem [12,13,16,28]. Additionally, a recent meta-analysis emphasized that health professionals need to assess patients’ internal needs and desires for behavioral changes and maintenance [14,29]. The evidence indicates that patients with chronic diseases need to recognize their role as a main decision-maker in managing their own health, and this recognition is crucial to activate autonomous motivation for healthy behaviors [14,15]. Our findings demonstrate that supportive interactions with health professionals could become a critical opportunity whereby patients recognize their ability to handle health issues, which could promote healthy behaviors. In particular, hypertensive patients with cardiovascular comorbidities are often faced with difficulties in dealing with multiple and ambiguous symptoms and signs, which may reduce their confidence to cope with their diseases. Therefore, health professionals’ autonomy-supportive attitudes should be highlighted as central to facilitate patients’ self-led behavioral regulation.

Some limitations of the current study need to be acknowledged. Data from this study were collected via self-administered surveys. Although self-reports are useful in socio-behavioral studies, they have potential disadvantages due to the risk of response bias, including social desirability bias and its covariates [30]. In addition, this study applied a cross-sectional design that limits the ability to confirm causality in the cognitive-behavioral processes of people with chronic diseases. Furthermore, the sample size of this study was relatively small, even though the statistical power was satisfactory to test the hypothesized model. Considering the increasing prevalence of cardiovascular comorbidities globally and the significant physical and psychosocial quality of life effects among the patients, a further study including larger samples is necessary.

Despite these limitations, it is notable that this study delineated a more comprehensive and specific cognitive-behavioral process by showing that autonomy support from health professionals plays a key role in determining the impact of personal control on behavioral regulation and psychological well-being.

## 5. Conclusions

The current study delineated the cognitive-behavioral mechanism by which personal control affects psychological well-being through healthy behaviors, based on differing levels of autonomy support from health professionals among hypertensive patients with cardiovascular comorbidities. Our findings demonstrated that autonomy support from health professionals moderated the role of healthy behaviors that mediate the relationship between personal control and psychological well-being, in terms of a moderated mediation effect.

The critical issues in this study are summarized as follows:

- Autonomy support from health professionals moderated the relationship between personal control and healthy behaviors. The relationship was more apparent among patients who experienced relatively higher support for autonomy from health professionals than those with lower or moderate levels of autonomy support.
- Further, autonomy support moderated the impact of personal control on psychological well-being through healthy behaviors. The role of healthy behaviors mediating the relationship between personal control and psychological well-being was found only in patients who had experienced higher support for autonomy from health professionals.
- The supportive attitudes of health professionals that facilitate the autonomy of patients are crucial to improve healthy behaviors and psychological well-being in patients. Health professionals should play a key role in encouraging patients’ autonomy and reinforcing their confidence in dealing with their disease through respectful interactions.

## Figures and Tables

**Table 1:** Sociodemographic and health-related characteristics of the participants (*N* = 149).

Characteristics	Categories	M ± SD or *n* (%)
Age		60.2 ± 9.0
Gender	Female	84 (56.4)
	Male	65 (43.6)
Education	High school graduation or under	95 (63.8)
	College graduates or beyond	54 (36.2)
Living status	Living with family	129 (86.6)
	Living alone	20 (13.4)
Job status	Currently employed	97 (65.1)
	Not employed	52 (34.9)
Monthly household income (Korean Won)	<2,000,000	59 (39.6)
	≥2,000,000	90 (60.4)
General health status		2.8 ± 0.8
Years since hypertension diagnosis		6.9 ± 6.6
Types of cardiovascular comorbidity	Hyperlipidemia	65 (43.6)
	Diabetes	33 (22.1)
	Abdominal obesity	28 (18.8)
	Diabetes and hyperlipidemia	23 (15.5)

**Table 2:** Descriptive statistics and correlations between the main study variables (*N* = 149).

	M	SD	1	2	3
1. Autonomy support	4.90	1.10	-		
2. Personal control	3.49	0.56	0.018	-	
3. Healthy behaviors	2.62	0.47	0.294 ***	0.145 ^†^	-
4. Psychological well-being	1.63	2.50	0.074	0.416 ***	0.239 **

*** *p* < 0.001, ** *p* < 0.01, ^†^ *p* < 0.10.

**Table 3:** Moderation effect of autonomy support on the relationship between personal control and healthy behaviors, and mediation effect of healthy behaviors on the relationship between personal control and psychological well-being.

Predictors	Healthy Behaviors			Psychological Well-Being		
	B	SE	*t*	B	SE	*t*
Constant	2.42 **	0.38	6.30	−4.29 *	2.10	−2.05
Personal control (I)	0.05	0.08	0.69	1.48 **	0.37	4.00
Autonomy support (M)	0.13 **	0.03	3.71			
Interaction between I and M	0.16 *	0.06	2.48			
Healthy behaviors				0.96 *	0.40	2.42
Covariates						
General health status	0.04	0.05	0.80	0.49 *	0.24	2.04
Years since HTN ^†^ diagnosis	−0.01 *	0.01	−2.15	−0.04	0.03	−1.10
Age	0.00	0.01	0.82	0.03	0.03	0.92
Job	−0.13	0.09	−1.37	1.13 *	0.45	2.48
	R^2^ = 19.78%, F(7, 135) = 4.75 **			R^2^ = 27.92%, F(6, 136) = 8.78 **		
	**An indirect effect of personal control on psychological well-being through healthy behaviors**					
The level of autonomy support	**B**		**Boot SE**	**LL 95% CI**		**UL 95% CI**
Low	−0.11		0.13	−0.392		0.116
Moderate	0.05		0.08	−0.097		0.238
High	0.22		0.12	0.018		0.475
	**A moderated mediation effect**					
Healthy behaviors	**Index**		**Boot SE**	**LL 95% CI**		**UL 95% CI**
	0.15		0.08	0.006		0.335

Note. Bootstrap samples = 5000, ^†^ HTN: Hypertension, ** *p* < 0.01, * *p* < 0.05.

## Data Availability

The data included in the present study are not publicly available to protect the confidentiality of the participants.

## References

[1] Zhou B., Carrillo-Larco R.M., Danaei G., Riley L.M., Paciorek C.J., Stevens G.A., Gregg E.W., Bennett J.E., Solomon B., Singleton R.K. (2021). Worldwide trends in hypertension prevalence and progress in treatment and control from 1990 to 2019: A pooled analysis of 1201 population-representative studies with 104 million participants. Lancet.

[2] Hosseinzadeh R., Goharrizi M.A.S.B., Bahardoust M., Alvanegh A.G., Ataee M.R., Bagheri M., Navidiyan E.S., Zijoud S.R.H., Heiat M. (2021). Should all patients with hypertension be worried about developing severe coronavirus disease 2019 (COVID-19)?. Clin. Hypertens..

[3] Phelps M., Christensen D.M., Gerds T., Fosbøl E., Torp-Pedersen C., Schou M., Køber L., Kragholm K., Andersson C., Biering-Sørensen T. (2020). Cardiovascular comorbidities as predictors for severe COVID-19 infection or death. Eur. Hear. J. Qual. Care Clin. Outcomes.

[4] Kim H.C., Cho S.M.J., Lee H., Lee H.-H., Baek J., Heo J.E., Ahn S.V., Jee S.H., Park S., Lee H.-Y. (2021). Korea hypertension fact sheet 2020: Analysis of nationwide population-based data. Clin. Hypertens..

[5] Chin Y.R., Lee I.S., Lee H.Y. (2014). Effects of Hypertension, Diabetes, and/or Cardiovascular Disease on Health-related Quality of Life in Elderly Korean Individuals: A Population-based Cross-sectional Survey. Asian Nurs. Res..

[6] Beck A.T. (2002). Cognitive models of depression. Clinical Advances in Cognitive Psychotherapy: Theory and Application.

[7] Skaff M.M. (2007). Sense of control and health. Handbook of Health Psychology and Aging.

[8] Náfrádi L., Nakamoto K., Schulz P.J. (2017). Is patient empowerment the key to promote adherence? A systematic review of the relationship between self-efficacy, health locus of control and medication adherence. PLoS ONE.

[9] Leventhal H., Phillips L.A., Burns E. (2016). The Common-Sense Model of Self-Regulation (CSM): A dynamic framework for understanding illness self-management. J. Behav. Med..

[10] Hagger M., Orbell S. (2003). A Meta-Analytic Review of the Common-Sense Model of Illness Representations. Psychol. Health.

[11] Ryan R.M., Deci E.L. (2000). Self-determination theory and the facilitation of intrinsic motivation, social development, and well-being. Am. Psychol..

[12] Warner L.M., Ziegelmann J.P., Schüz B., Wurm S., Tesch-Römer C., Schwarzer R. (2011). Maintaining autonomy despite multimorbidity: Self-efficacy and the two faces of social support. Eur. J. Ageing.

[13] Ryan R.M., Patrick H., Deci E.L., Williams G.C. (2008). Facilitating health behaviour change and its maintenance: Interventions based on self-determination theory. Eur. Health Psychol..

[14] Ntoumanis N., Ng J.Y., Prestwich A., Quested E., Hancox J.E., Thøgersen-Ntoumani C., Deci E.L., Ryan R.M., Lonsdale C., Williams G.C. (2021). A meta-analysis of self-determination theory-informed intervention studies in the health domain: Effects on motivation, health behavior, physical, and psychological health. Heath Psychol. Rev..

[15] Kayser J.W., Cossette S., Alderson M. (2013). Autonomy-supportive intervention: An evolutionary concept analysis. J. Adv. Nurs..

[16] Su Y.-L., Reeve J. (2010). A Meta-analysis of the Effectiveness of Intervention Programs Designed to Support Autonomy. Educ. Psychol. Rev..

[17] Faul F., Erdfelder E., Lang A.-G., Buchner A. (2007). G*Power 3: A flexible statistical power analysis program for the social, behavioral, and biomedical sciences. Behav. Res. Methods.

[18] Cohen J. (2013). Statistical Power Analysis for the Behavioral Sciences.

[19] Moss-Morris R., Weinman J., Petrie K., Horne R., Cameron L., Buick D. (2002). The Revised Illness Perception Questionnaire (IPQ-R). Psychol. Health.

[20] Williams G.C., Grow V.M., Freedman Z.R., Ryan R.M., Deci E.L. (1996). Motivational predictors of weight loss and weight-loss maintenance. J. Personal. Soc. Psychol..

[21] Song R., June K.J., Kim C.G., Jeon M.Y. (2004). Comparisons of Motivation, Health Behaviors, and Functional Status among Elders in Residential Homes in Korea. Public Health Nurs..

[22] Song H.-Y., Nam K.A. (2015). Effectiveness of a Stroke Risk Self-Management Intervention for Adults with Prehypertension. Asian Nurs. Res..

[23] Mechanic D., Bradburn N.M. (1970). The Structure of Psychological Well-Being. Am. Sociol. Rev..

[24] Hayes A.F. (2017). Introduction to Mediation, Moderation, and Conditional Process Analysis: A Regression-Based Approach.

[25] Hagger M.S., Chatzisarantis N. (2009). Integrating the theory of planned behaviour and self-determination theory in health behaviour: A meta-analysis. Br. J. Health Psychol..

[26] Arija V., Villalobos F., Pedret R., Vinuesa A., Jovani D., Pascual G., Basora J. (2018). Physical activity, cardiovascular health, quality of life and blood pressure control in hypertensive subjects: Randomized clinical trial. Health Qual. Life Outcomes.

[27] Xu H., Deng K., Lin Z., Huang Z., Gong X., Tan J., Huang B., Gao Y. (2020). The effects of physical activity and sedentary behavior in the associations between cardiovascular diseases and depression: A four-way decomposition. J. Affect. Disord..

[28] Senécal C., Nouwen A., White D. (2000). Motivation and dietary self-care in adults with diabetes: Are self-efficacy and autonomous self-regulation complementary or competing constructs?. Health Psychol..

[29] Lee A.A., Piette J.D., Heisler M., Janevic M.R., Rosland A.-M. (2019). Diabetes self-management and glycemic control: The role of autonomy support from informal health supporters. Health Psychol..

[30] Podsakoff P.M., MacKenzie S.B., Podsakoff N.P. (2012). Sources of method bias in social science research and recommendations on how to control it. Annu. Rev. Psychol..

